# Differences in Perceived Mental Effort Required and Discomfort during a Working Memory Task between Individuals At-risk And Not At-risk for ADHD

**DOI:** 10.3389/fpsyg.2017.00407

**Published:** 2017-03-21

**Authors:** Chia-Fen Hsu, John D. Eastwood, Maggie E. Toplak

**Affiliations:** ^1^Department of Psychology, Faculty of Health, York University, TorontoON, Canada; ^2^Department of Psychology, Chung Shan Medical UniversityTaichung, Taiwan; ^3^Clinical Psychological Room, Chung Shan Medical University HospitalTaichung, Taiwan

**Keywords:** experience of effort, mental effort, subjective ratings, ADHD-risk, cognitive abilities

## Abstract

**Objective:** The avoidance of mental effort is a symptom criterion for Attention-Deficit/Hyperactivity Disorder (ADHD), but the experience of mental effort has received relatively little attention in the empirical study of individuals at-risk for ADHD. We explored a novel method to assess the experience of effort and discomfort during a working memory task in a sample of young adults at-risk and not at-risk for ADHD.

**Method:** A sample of 235 undergraduate students (Mean age = 21.02, 86 males) were included in this study. Based on an ADHD-screener (ASRS), 136 participants met criteria for the ADHD-risk group and 99 were in the non-ADHD risk group.

**Results:** Individuals at-risk for ADHD reported higher mental effort and discomfort than individuals not at-risk for ADHD, even when performance on the working memory task was comparable or statistically controlled. Mental effort required and discomfort were more strongly correlated for at-risk compared to not at-risk participants. Individuals at-risk for ADHD displayed a stronger correlation between mental effort required and actual accuracy, but individuals not at-risk for ADHD displayed a stronger association between perceived accuracy and actual accuracy for the hardest experimental conditions. The most intense moment of effort required predicted retrospective discomfort ratings of the task in the ADHD-risk group, but not in the non-risk group.

**Conclusion:** The subjective experience of *in the moment* mental effort is an important and viable construct that should be more carefully defined and measured. In particular, the experience of effort required (or how taxing a task is) differentiated between individuals at-risk and individuals not at-risk for ADHD in the present study. Whereas previous ADHD research has explored effort exerted, the present work demonstrated that investigating the experience of being mentally taxed might provide a productive line of investigation that could be used to advance our understanding of the cognitive and affective mechanisms underlying the regulation of effort in individuals at-risk of ADHD.

## Introduction

The point of departure for the present work is the observation that there are individual differences in how mentally effortful tasks are experienced. For some, a cognitively demanding task is a welcomed opportunity, for others such a task can’t end soon enough. Individual differences are evident in both what it feels like *‘in the moment’* to complete a mentally effortful task (e.g., *this* task is requiring a lot of effort; see for example, Paas, 1992; von Helversen et al., 2008; Robinson and Morsella, 2014) and *‘generalized thoughts and feelings’* about mental effort (e.g., I often find math questions require a lot of effort; see for example, Dornic et al., 1991; Cacioppo et al., 1996). This difference between effort in the moment and generalized thoughts and feelings largely maps onto the classic distinction between states and traits. In this study, we focused on measuring the “*in the moment*” experience of effort during a cognitively demanding task. In addition, we identified individuals at-risk for Attention-Deficit/Hyperactivity Disorder (ADHD), as avoidance of effortful tasks is diagnostic of these individuals (DSM-5, American Psychiatric Association [APA], 2013). We examined differences between those at-risk for ADHD and those not at-risk in terms of their experience of mental effort during a cognitive demanding task, and whether these differences were separable from task performance.

The *‘in the moment’* versus *‘generalized thoughts and feelings’* about mental effort distinction provides some conceptual clarity for understanding the experience of mental effort, but there is still a long way to go in defining this construct in a more precise manner for proper empirical investigation. In part, this is because the experience of mental effort is a broad and often ill-defined concept with a variety of different aspects. Like the proverbial blind men, researchers have grasped different aspects of the elephant and come to different understandings. There are at least three distinct aspects of mental effort that need to be distinguished in participants’ subjective self-reports. Participants can report *‘how hard I tried’* (i.e., effort volitionally exerted); *‘how taxed I felt’* (i.e., effort extracted for level of achievement) and *‘how difficult the task is’* (i.e., effort potentially demanded). The key difference between *‘how hard I tried’* and *‘how taxed I felt’* relates to agency. The first puts the emphasis on what one brings to the task; that is, one’s level of motivation and energy to do one’s best, or not. Whereas the second puts the emphasis on what the task does to the individual; that is, how much the task burdened or required of the individual. It is possible to imagine situations where a person might report trying hard to do their best but also report finding that the task did not require a lot of them. This conceptual distinction has borne out empirically. For example, Mulert et al. (2007) found that amount of “effort required” (i.e., how taxed I felt) varied in direct proportion to objective task difficulty, whereas amount of “volitional effort” (i.e., how hard I tried) was more constant across variations in task difficulty. The key difference between *‘how taxed I felt’* and *‘how difficult the task is’* is whether the focus is on the burden experienced by the individual or on the external task and its characteristics. The first puts the emphasis on what the task does to the individual (*how much the task burdened or required of me*), whereas the second puts the emphasis on characteristics of the task (*whether it is difficult relative to other possible tasks*). It is possible to imagine situations where a person might report feeling very taxed and report that the task was not very difficult; perhaps they did not get a good night of sleep and are thus very inattentive, but recognize that relative to other tasks this one is actually not that difficult. This conceptual distinction has also been borne out empirically. For example, Otto et al. (2014) found that the ratings of mental effort (i.e., *how taxed I felt*) were associated with increased activation in the left anterior insular cortex (aIC) compared to ratings of task difficulty (i.e., *how difficult the task is*) during a working memory task. Taken as a whole, there are conceptual and empirical reasons to believe that investigators studying mental effort must take care to distinguish between these different aspects of the experience of mental effort.

Mental effort is central to the definition of ADHD, surprisingly, however, this topic has garnered relatively little systematic research. One of the ADHD criterion stipulates that an individual with ADHD: “often avoids, dislikes, or is reluctant to engage in tasks that require sustained mental effort (e.g., schoolwork or homework; for older adolescents and adults, preparing reports, completing forms, reviewing lengthy papers).” (p. 59, American Psychiatric Association [APA], 2013). Given the requirement for pervasiveness of signs and symptoms in order to obtain a diagnosis of ADHD, this diagnostic criterion suggests a dispositional quality related to avoidance of effort in individuals with ADHD. The Brown Scale is a standardized tool that was developed to provide a broader clinical characterization of the inattentive symptoms and executive problems associated with ADHD (Brown, 1996). This measure includes an Effort Scale, defined by inconsistent energy and inconsistent sustained effort. It appears that the Effort Scale is assessing something akin to the ‘*how hard I (typically) try*’ aspect of mental effort given that items suggest a lack of energy and a failure to work to potential. Adolescents with ADHD have reported more difficulty sustaining effort as indicated by significant differences on the Effort Scale compared to an adolescent control group (Rucklidge and Tannock, 2002). Similarly, the Cognitive and Motivation in Everyday Life (CAMEL) Scale (Van Liefferinge et al., 2016), which assesses neuropsychological impairments in children and youth with ADHD, appears to tap into the *‘how hard I (typically) try’* aspect of mental effort. Sample items from the Effort Allocation Scale of the CAMEL are: “Takes time to warm-up – to get going on a task”; “Puts things off until the last minute,” and “Chooses the way with least effort.” Parents were asked to rate their children and youth based on the extent to which the items described their children’s behavior using a five-point Likert scale. Children with ADHD scored significantly lower on this scale compared to children in the community sample, demonstrating a medium effect size, indicating difficulties with effort allocation in the clinical group.

Sergeant’s (2000, 2005) cognitive-energetic model has proposed a role for state effort in explaining ADHD deficits. In this model, effort is conceptualized as a state factor (including arousal and activation), which is separable from computational mechanisms (such as encoding, search, decision, and motor organization) and management or evaluation mechanisms (related to executive functions). Specifically, effort is defined as “the necessary energy to meet the demands of a task” (Sergeant, 2000, p. 8). In this model, task demands are thought to impact effort, whereby greater cognitive load elicits greater mobilization of energy (or effort). Relatively little research has examined the energetic state component of Sergeant’s model. However, children with ADHD have been shown to perform more poorly in tasks with slow compared to fast event rates (Sergeant et al., 1999). Unlike previous research on ADHD and mental effort, Sergeant’s model focuses on state *‘in the moment’* effort. But like previous ADHD research, this model appears to be focusing on the *‘how hard I tried’* aspect of mental effort. That is, suggesting that individuals with ADHD struggle to mobilize and volitionally exert the effort needed, especially on tasks that are de-energizing.

Given that avoidance of mentally effortful tasks is a diagnostic criterion of ADHD, it is somewhat surprising that relatively little systematic research has been done on the topic. Most of the research has focused on ratings of general thoughts, feelings and dispositions and even more specifically trait dispositions regarding how hard individuals try to complete mentally effortful tasks. A smaller amount of work, largely centered around Sergeant’s theory, has modeled a key role for *in the moment* considerations focusing on how much effort is exerted. To our knowledge, no research has examined whether individuals with ADHD or related difficulties systematically differ in their experience of ‘how taxed’ they are by cognitive tasks compared to non-ADHD individuals. Given that many theories of mental effort postulate that the experience of being mentally taxed may serve as a regulatory signal to disengage from effortful tasks (e.g., Kurzban, 2016), investigating the experience of being mentally taxed in individuals with ADHD-related difficulties may prove to be a productive focus of investigation that could be used to advance our understanding of the regulation of effort in at-risk samples. Thus, the overarching goal of the present work was to systematically describe how the subjective experience of mental effort required (i.e., experience of being burdened or taxed) is different for individuals at-risk for ADHD compared to those not at-risk for ADHD.

In the current study, we modeled our experimental design and data analytic approach on a pain study completed by Redelmeier and Kahneman (1996). During colonoscopy and lithotripsy medical procedures (which were painful procedures at the time their study was conducted), Redelmeier and Kahneman (1996) asked participants to report their level of pain every 60 s. Then, within 1 h of completing the medical procedure, participants were asked to recall the total amount of pain they experienced. These authors found that the most intense ratings of pain during the procedure and the final ratings of pain at the end of the procedure were the most robust predictors of retrospective ratings – this phenomenon has come to be called the ‘peak-end effect.’ In a review of research on the ‘peak-end’ effect, Fredrickson (2000) argued that the broader principle is that moments of personal significance or salience are the best predictors of recall, and that peak and final moments are typically salient for most people. Applying this methodology from the pain literature, we assessed how taxed and uncomfortable participants felt during the completion of a cognitively demanding task and how uncomfortable participants recall the task to be after it was completed. We also measured their actual performance and their perceived performance.

We examined whether individuals at-risk for ADHD would find a cognitively demanding task more taxing (i.e., effort required) and uncomfortable than individuals not at-risk for ADHD. Specifically, we used an *in the moment* methodology that provides a specific reference point to a cognitively demanding task for these ratings. We asked participants to rate the effort required and discomfort at regular intervals during this task. We predicted that the at-risk group would rate the task as requiring more effort and as more uncomfortable than the non-risk group, even when performance accuracy was comparable in both groups. Further, if effort required and discomfort is more salient in the at-risk group these ratings should be more strongly associated with performance accuracy among the at-risk group than in the non-risk group. Finally, given our predictions regarding the salience of effort required and discomfort in the at-risk group, we predicted that the most intense (or peak ratings) of effort required would be particularly strong predictors of the at-risk group’s retrospective memories of discomfort associated with the working memory task. Given the novelty of the methodology used in this study, we examined our hypotheses in extreme groups that were identified using cut-offs on an ADHD symptom screening measure (at-risk ADHD and non-risk ADHD groups).

## Materials and Methods

### Ethics Statement

The experimental protocol was approved by the Human Participants Review Committee of York University’s Office of Research Ethics. Written, informed consent was obtained from all participants at the very beginning of the research protocol before any data was collected. Participants were free to withdraw consent at any time and still receive course credit.

### Participants

A sample of 235 participants (Mean age = 21.02, *SD* = 5.69, 86 males) was selected for inclusion in this study from a larger sample of 431 university undergraduate students (mean age: 20.59, *SD*: 4.97, age range: 17–55, female: 68%) who participated in a larger study for course credit. Data from 30 participants were excluded from the analyses for the following reasons: participants failed the practice trials on the working memory task, participants were deemed to be outliers due to excessively poor performance on the working memory task (i.e., accuracy at any block was more than three standard deviations away from the mean of the blocks with the same difficulty) or participants had incomplete or missing data. Some data from these participants are reported in another study addressing different questions (Hsu et al., unpublished).

In the present study, we used the adult ADHD Self-Report Symptom Checklist screener (ASRS, Kessler et al., 2005) to identify extreme groups, so that we could compare participants with ADHD-related difficulties to participants who are not at-risk. Namely, participants who scored above the threshold on four or more items were identified as at-risk of ADHD, and participants who score above the threshold on one or zero items were classified as not at-risk. Based on this ASRS screening criterion, we selected 136 at-risk participants (mean age: 20.49, *SD* = 5.20, 49 males) and 99 not at-risk participants (mean age: 21.77, *SD* = 6.26, 37 males) for the ADHD non-risk group resulting in a total of 235 participants for final analysis. The two groups did not differ in age and gender (all *p* > 0.05).

### Measures

#### Working Memory Task

We used an adapted version of the Paced Auditory Serial Addition Test (PASAT, Gronwall, 1977) to elicit mental effort. In this task digits were visually presented on a computer screen, one at a time, in a continuous stream. Participants were asked to continuously add the numerical digits. Each digit was presented on the screen for 1 s. An orienting auditory beep was simultaneously presented with each digit. The delay between the offset of one digit and the onset of the next digit was 4 s. Participants were instructed to sum the last two digits they had seen and to enter their response using a keyboard. Participants were able to respond as soon as the latest digit appeared and during the 4 s delay; thus, within a response window of 5 s. Each ‘block’ required participants to provide a total of 15 sums (i.e., 15 trials with a total of 16 digits presented). The task had five conditions differing in duration, i.e., the number of blocks ranging from five to nine blocks. Participants were randomly assigned to one of five conditions. Each block was one of three levels of difficulty – easy, medium or hard. Task difficulty was randomly varied within participants such that each participant received one easy block, one hard block, and three to seven medium difficulty blocks depending on the duration condition completed. The blocks were presented in a pseudo-randomized sequence, such that the first and last blocks were always medium difficulty. The intervening blocks were composed of one easy, one hard, and one to five medium blocks. The hard trials involved adding one single and one double digit. The medium trials involved adding two single digits that summed to more than nine. The easy trials involved adding two single digits that summed to nine or less.

At the start of the PASAT participants completed two practice blocks of medium difficulty to ensure they fully comprehended the task and to familiarize them with the task demands. Following the practice blocks, participants were asked some questions regarding their anticipated experience of the task (including how much mental effort they expected would be required to complete the task and how much discomfort they expected during the task; the data on these questions were not examined in this study). After each block of trials, participants were required to rate their experience of effort and discomfort. Specifically, the questions were (1) “Rate your current level of mental effort” (1 = None; 7 = A lot) and (2) “Rate your current level of discomfort or distress” (1 = None; 7 = A lot). These scores are referred to as ‘real-time’ mental effort and discomfort ratings as they were obtained during the PASAT. After completing the PASAT task, participants completed a demographic and self-report questionnaire that was approximately 6 min in duration. After completing the demographic questionnaire, participants were required to retrospectively evaluate their experience with the PASAT task. Specifically, the questions included (1) “How much mental effort, in total, was required to complete this working memory task?” (1 = None; 7 = A lot); (2) “On this working memory task, what was your total amount of discomfort or distress?” (1 = None; 7 = A lot); (3) “How well did you perform on the working memory task” (1 = Significantly below average; 7 = Significantly above average); and (4) “How willing would you be to do another working memory task right now?”(1 = Not at all willing; 7 = Definitely willing).

#### Raven’s Progressive Matrices

Advanced items from the Raven’s Progressive Matrices (RPM) were used to assess participants’ non-verbal intelligence (Raven et al., 1998). This task contains a series of designs with a missing part. Participants were instructed to complete the design by selecting the missing part from eight options. This task had 18 trials in total and a time limit of 15 min. The number of correct responses was summed such that a higher score indicated higher non-verbal ability.

#### ADHD Self-Report Scale

The ADHD self-report scale (ASRS) screener was developed by Kessler et al. (2005) and is based on symptoms of ADHD described in the Diagnostic and Statistical Manual of Mental Disorders (fourth edition). Participants are asked to rate their level of each symptom based on the last 6 months. Of the 18 symptoms, Kessler et al. (2005) found six items to be highly predictive of ADHD, and these six items comprise the screener. Within the six items of the screener, four are based on symptoms of inattention with the other two based on hyperactivity. Sample question includes: “*How often do you have problems remembering appointments or obligations?.*” It is scored using a five-point Likert scale (never, rarely, sometimes, often, and very often). Cronbach’s alpha for this scale was 0.73. Each item has a frequency threshold, and participants who scored above the threshold on four or more items were identified as the ADHD-risk group and participants who scored above the threshold on one or zero items were identified as the ADHD non-risk group.

### Statistical Analyses

In order to examine differences between the ADHD-risk and non-risk groups, we used independent *t*-tests to examine differences in: real-time ratings of mental effort and discomfort, task performance, slopes of real-time mental effort and discomfort and non-verbal ability. Pearson’s correlation analyses were used to examine the relationship between real-time effort and discomfort and between the average of real-time mental effort, discomfort, perceived performance and actual performance on the PASAT separately in the ADHD-risk and non-risk groups. The group differences in correlations were examined using Fisher’s *r*-to-*z* transformation. Finally, the association between effort (peak and end) and retrospective discomfort were examined separately in the ADHD-risk and non-risk groups, including regression analyses to examine whether the most intense ratings of effort interacted with ADHD status in predicting retrospective ratings of discomfort.

## Results

### Did the ADHD-Risk and ADHD Non-risk Group Differ in Their Experience of Mental Effort?

As evident in **Table 1**, the ADHD-risk group experienced a higher level of mental effort and discomfort during the PASAT. As for the retrospective evaluation, the ADHD-risk group recalled experiencing a higher level of mental effort and discomfort compared to the non-risk group. The ADHD-risk group also reported a significantly lower level of perceived performance on the PASAT, and was more reluctant to repeat the task compared to the non-risk group. The ADHD-risk group performed significantly worse than the non-risk group on the PASAT, especially on the hard and medium blocks of PASAT. There was no group difference in accuracy on the easy block. Effect sizes for each of the comparisons are presented in the final column, indicating medium to large effect sizes on several of the group differences related to mental effort and discomfort.

**Table 1:** Differences between the Attention-Deficit/Hyperactivity Disorder (ADHD) non-risk and ADHD-risk groups.

	Control *n* = 99 Mean (*SD*)	ADHD-risk *n* = 136 Mean (*SD*)	*t* (233)	Cohen’s *d*
**Real-time effort**				
Average	4.23 (1.18)	4.88 (1.26)	-4.00^∗∗∗^	0.53
Hard block	5.02 (1.48)	5.64 (1.39)	-3.29^∗∗∗^	0.43
Medium block	4.15 (1.23)	4.80 (1.30)	-3.86^∗∗∗^	0.51
Easy block	3.83 (1.50)	4.46 (1.75)	-2.99^∗∗^	0.39
Peak	5.44 (1.20)	6.00 (1.15)	-3.60^∗∗∗^	0.48
End	4.05 (1.54)	4.74 (1.73)	-3.22^∗∗^	0.42
**Real-time discomfort**				
Average	3.11 (1.45)	4.15 (1.58)	-5.15^∗∗∗^	0.69
Hard block	3.72 (1.84)	4.89 (1.88)	-4.77^∗∗∗^	0.63
Medium block	3.04 (1.47)	4.06 (1.59)	-5.02^∗∗∗^	0.67
Easy block	2.79 (1.54)	3.76 (1.95)	-4.26^∗∗∗^	0.55
Peak	4.19 (1.89)	5.33 (1.74)	-4.78^∗∗∗^	0.63
End	3.07 (1.58)	4.12 (1.99)	-4.33^∗∗∗^	0.58
**PASAT accuracy**				
Average	0.79 (0.16)	0.74 (0.15)	2.38^∗^	0.32
Hard block	0.66 (0.25)	0.58 (0.23)	2.43^∗^	0.33
Medium block	0.80 (0.17)	0.75 (0.16)	2.12^∗^	0.30
Easy block	0.85 (0.17)	0.81 (0.20)	1.27	0.22
**Slope**				
Real-time effort (Z)	-0.03 (0.23)	-0.04 (0.30)	0.37	0.04
Real-time discomfort (Z)	0.03 (0.26)	0.02 (0.32)	0.23	0.03
**Retrospective ratings**				
Remembered effort	4.53 (1.42)	5.35 (1.28)	-4.67^∗∗∗^	0.61
Remembered discomfort	3.38 (1.54)	4.60 (1.74)	-5.54^∗∗∗^	0.74
Perceived performance	4.47 (1.20)	3.79 (1.35)	-4.04^∗∗∗^	0.53
Willing to repeat task	3.86 (1.92)	3.17 (2.02)	2.64^∗∗^	0.35
**Non-verbal ability**				
Non-verbal ability	5.88 (2.80)	5.88 (3.40)	-0.01	0

*^∗^*p* < 0.05; ^∗∗^*p* < 0.01; ^∗∗∗^*p* < 0.001.*

We conducted a univariate ANOVA to examine whether group differences on both effort required and discomfort ratings (and separately for real-time and retrospectively) would remain significant after statistically controlling for mean performance. Group differences remained significant for both real-time effort required, *F*(1,232) = 12.99, *p* < 0.001, and retrospective effort required, *F*(1,232) = 18.89, *p* < 0.001. Group differences also remained significant for both real-time discomfort, *F*(1,232) = 21.99, *p* < 0.001, and retrospective discomfort, *F*(1, 232) = 26.46, *p* < 0.001.

The difference in the slopes of real-time mental effort and discomfort between groups was non-significant, suggesting that the change of effort and discomfort over time were comparable between the two groups. There was also no significant difference in non-verbal ability for the ADHD-risk group and the non-ADHD risk group.

In addition, we examined group differences in the magnitude of the correlations between real-time effort and real-time discomfort ratings. The size of this correlation was *r* = 0.43, *p* < 0.001 in the non-risk group and the size of this correlation was *r* = 0.62, *p* < 0.001 in the ADHD-risk group. The correlation between real-time mental effort and discomfort was significantly higher within the ADHD-risk group as compared to the non-risk group (Fisher’s *Z* = 1.98, *p* < 0.05).

### Does the Relation between Subjective Experience and Task Performance Differ between ADHD Non-risk and ADHD-risk Groups?

**Table 2** compares correlations between average real-time mental effort required and task performance for the ADHD-risk and non-risk groups. The ADHD-risk group showed significant correlations between average real-time ratings of mental effort required and performance accuracy on the PASAT, indicating that more effort required was associated with lower accuracy. Alternatively, the non-risk group did not show significant correlations between average real-time mental effort required and performance accuracy. The magnitude of the correlation between average real-time mental effort and PASAT accuracy in the hard condition was significantly larger in the ADHD-risk group compared to the non-risk group based on a one-tailed *Z* test, Fisher’s *Z* (1, *N* = 235) = 1.78, *p* < 0.05. Thus, taken together, the relation between real-time mental effort required and task performance appears to be more robust for the ADHD-risk group than in the non-risk group, especially in the most difficult condition. We obtained parallel findings when we examined associations between ratings of discomfort and performance accuracy, and this difference between groups was also statistically significant in the most difficult condition, Fisher’s *Z* (1, *N* = 235) = 1.79, *p* < 0.05. We also examined the association between perceived performance and actual performance in each group. Both groups displayed significant positive correlations, but the correlation for the non-risk group was significantly larger than for the ADHD-risk group in the difficult condition, Fisher’s *Z* (1, *N* = 235) = 1.69, *p* < 0.05.

**Table 2:** Correlations between average real-time mental effort and cognitive performance for the ADHD-risk and ADHD non-risk groups.

	ADHD non-risk *n* = 99 Mean *(SD)*	ADHD-risk *n* = 136 Mean *(SD)*	*Fisher’s* Z
**Real-time average effort and actual accuracy**			
Effort ( 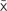 ) and actual accuracy ( 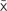 )	-0.12	-0.20*	0.61
Effort ( 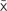 ) and actual accuracy (hard)	-0.06	-0.29***	1.78^∗^
Effort ( 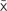 ) and actual accuracy (medium)	-0.15	-0.17	0.15
Effort ( 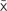 ) and actual accuracy (easy)	-0.01	-0.05	0.30
**Real-time average discomfort and actual accuracy**			
Discomfort ( 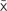 ) and actual accuracy ( 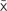 )	-0.14	-0.25*	0.85
Discomfort ( 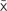 ) and actual accuracy (hard)	-0.08	-0.31***	1.79^∗^
Discomfort ( 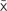 ) and actual accuracy (medium)	-0.17	-0.19*	0.15
Discomfort ( 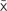 ) and actual accuracy (easy)	-0.03	-0.21*	1.37
**Perceived accuracy and actual accuracy**			
Perceived accuracy and actual accuracy ( 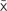 )	0.39^∗∗∗^	0.24**	1.25
Perceived accuracy and actual accuracy (hard)	0.49^∗∗∗^	0.30***	1.69^∗^
Perceived accuracy and actual accuracy (medium)	0.36^∗∗∗^	0.22*	1.14
Perceived accuracy and actual accuracy (easy)	0.13	0.07	0.45

*Asterisk indicated the significance of correlation coefficients within each group, where ^∗^*p* < 0.05; ^∗∗^*p* < 0.01; ^∗∗∗^*p* < 0.001. Two-tailed Z-critical is 1.96 for *p* < 0.05; One-tailed Z-critical is 1.65 for *p* < 0.05.*

### Do Peak and End Real-time Mental Effort Differentially Predict Retrospective Discomfort in the ADHD-risk and Non-risk Groups?

**Table 3** presents the results of two simultaneous multiple regression analyses using peak and end real-time effort required predicting retrospective discomfort, one conducted in the ADHD non-risk sample and the other conducted in the ADHD risk sample. While the overall models were significant for both groups, the peak real-time effort required was a significant predictor in the ADHD-risk group but not in the non-risk group.

**Table 3:** Simultaneous multiple regression analyses predicting the retrospective discomfort with real-time measures of effort in ADHD non-risk and ADHD-risk samples.

DV: Retrospective discomfort	Regression #1 ADHD non-risk (*n* = 99) β′	Regression #2 ADHD-risk (*n = 136*) β′
Peak real-time effort	0.22	0.45^∗∗∗^
End real-time effort	0.25^∗^	0.21^∗^
	Adjusted *r*^2^ = 0.16^∗∗∗^	Adjusted *r*^2^ = 0.35^∗∗∗^

*^∗^*p* < 0.05; ^∗∗∗^*p* < 0.001.*

**Table 4** presents the result of a simultaneous multiple regression analysis using ADHD group status, peak effort required and the interaction between ADHD group status and peak effort required predicting retrospective discomfort. The result showed that ADHD group status (β = 0.24, *p* < 0.001), peak effort required (β = .33, *p* < 0.001), and the interaction (β = .20, *p* < 0.05) were significant predictors. The interaction between group status and peak effort required indicates that the most intense moment of effort required was particularly salient in defining retrospective discomfort for those at-risk for ADHD.

**Table 4:** Simultaneous multiple regression analysis predicting the retrospective discomfort with real-time effort, ADHD group status and peak effort by ADHD group status.

DV: Retrospective discomfort	Whole sample (*n* = 235) β′
ADHD group status	0.24^∗∗∗^
Peak real-time effort	0.33^∗∗∗^
Peak real-time effort by ADHD group status	0.20^∗^
	Adjusted *r*^2^ = 0.34^∗∗∗^

*^∗^*p* < 0.05; ^∗∗∗^*p* < 0.001.*

### Length of Task

We examined the correlation between length of the task (number of blocks) with each of our variables, including accuracy and ratings of effort and discomfort, both in the full sample and within each group. We found that length of task did not systematically vary with any of these variables in the full sample, in the control group or in the ADHD-risk group.

## Discussion

In the current study, we examined individual differences in the experience of mental effort. Specifically, we asked participants at-risk for ADHD and participants not at-risk for ADHD to rate effort required and discomfort during a cognitively demanding task. We found that the ADHD-risk group reported higher levels of mental effort required (i.e., how taxed they felt) and discomfort during the PASAT working memory task, and these differences remained significant even after controlling for task performance. Individuals at-risk for ADHD were also less willing to do the working memory task again compared to the non-ADHD risk group.

The positive correlations observed between real-time ratings of effort and discomfort in both the ADHD-risk and control non-risk groups are consistent with the idea that the experience of effort is unpleasant and aversive (Stanovich, 2009, 2011; Kahneman, 2011; Kurzban et al., 2013). Researchers have suggested that effortful tasks tend to trigger an unpleasant, aversive state because human cognitive system naturally seeks to minimize the expenditure of effort (Kurzban et al., 2013). In this sense cognitive demands are evaluated as being costly and typically trigger avoidance coping behaviors (Botvinick, 2007). Importantly, however, the experience of effort required was more strongly associated with discomfort for individuals at-risk for ADHD than for the non-risk group, as indicated by the significant difference in the size of the correlations between effort and discomfort ratings. Moreover, we found that average real-time effort and retrospective effort differed significantly in the at-risk and non-risk groups even after controlling for task performance. These findings are consistent with the theoretical literature in suggesting that the experience of a cognitively demanding task may be especially uncomfortable and effortful for individuals who struggle with ADHD-related difficulties (Brown, 1996; Sergeant, 2000; Rucklidge and Tannock, 2002; Van Liefferinge et al., 2016). In summary, individuals at-risk for ADHD experienced the working memory task to require more effort and to be more uncomfortable, even after controlling for task performance; and they also found the experience of effort and discomfort to be more strongly correlated.

The ADHD-risk group showed significant correlations between average real-time ratings of mental effort required and accuracy, but the non-risk group did not display significant correlations between average real-time effort required and performance accuracy. Similarly the ADHD risk group showed significant correlations between average real-time ratings of discomfort and accuracy, but the non-risk group did not display significant correlations between discomfort and accuracy. These differences between the at-risk and not at-risk groups were most pronounced for the hardest experimental conditions. Namely, in the hardest experimental conditions, the experience of effort required and discomfort were more tightly associated with actual performance accuracy for the at-risk group compared to the non-risk group. In contrast, in the hardest experimental conditions the perception of performance and actual accuracy were more tightly associated for the non-risk group compared to the at-risk group. Taken together, these results suggest the best subjective report for predicting actual performance on particularly challenging working memory tasks is perceived performance for the non-risk participants; whereas the at-risk participants are less accurate in their perception of how well they are doing. For the at-risk group, perceptions of performance and the experience of effort and discomfort were similarly related to actual accuracy. These sets of associations suggest that some type of moment-to-moment tracking of actual performance is happening during the task, but that what is being tracked may differ across groups. These intercorrelations among the effort, discomfort, perceived performance, and actual accuracy in each group may provide some further insights into understanding the differences between each group.

Peak real-time effort required was a significant predictor of retrospective discomfort in the ADHD-risk group but not in the non-risk group. Moreover, the interaction between ADHD group status and peak effort predicted retrospective discomfort, suggesting that the ADHD-risk group’s most intense experience of effort required was highly related to their memory of discomfort. That peak effort required interacted with ADHD-risk group status suggests that moments of peak effort are particularly significant and salient for individuals at-risk for ADHD and thus may be particularly defining for these individuals when they recall tasks (see review by Fredrickson, 2000). We, however, did not find evidence to suggest that the final moments of a cognitively demanding task are differentially salient in determining task recollection for individuals at-risk of ADHD compared to those not at-risk. It may be that endings are equally salient for those at-risk for ADHD compared to those that are not at-risk.

In our view it will be fruitful to explore the *in the moment* experience of mental effort in a more nuanced manner, which disentangles subjective judgments of effort demanded (*how taxed I felt)*, effort exerted (*how hard I tried)*, and task difficulty (*how difficult the task is)*. In particular, these distinctions will be useful to extend the ADHD literature, which has so far focused more on trait measures (Brown, 1996; Van Liefferinge et al., 2016) and on effort exerted in the moment (Sergeant, 2000, 2005). We hope that our study provides a reference point for the utility of these distinctions in the experience of effort. In particular, the current findings suggest that individuals at-risk for ADHD may be characterized by differences in how taxed they feel during the completion of a cognitively demanding task, which is meaningfully separate from ratings of task difficulty and how hard one tries on a given task. Our research contributes to shifting emphasis from biomedical models that focus on the pathophysiology of disorders such as ADHD and to models that focus on the subjective experience and personal agency of the individual (e.g., Sonuga-Barke and Fairchild, 2012). Similarly, acknowledging the value and diagnosticity of an individual’s report of how demanding the task was is consistent with such a direction.

It is important to acknowledge that our study was based on a sample of university students, identifying at-risk and non-risk for ADHD groups. The proportion of at-risk individuals in our current study was quite high (31.6%) and considerably higher than prevalence rates of ADHD, which is closer to rates of 11% (DSM-5, American Psychiatric Association [APA], 2013) or 12% (Kessler et al., 2007). However, our proportion of at-risk participants is in line with other studies that have used the ASRS as a screening tool in undergraduate samples for ADHD symptoms (24% such as Turel and Bechara, 2016). We acknowledge that this is a limitation of our study and that future studies will be needed in order to bridge our current findings with the ADHD literature. A critical step will be to examine these measures in a clinically referred sample of children, adolescents and adults with diagnosed ADHD. Studying the experience of mental effort will provide novel ways to assess the challenges of these individuals, and to develop new directions to assess and intervene for these individuals. The more precise language for describing the ways that tasks impact the individual, which we are advocating here has the promise to help develop better strategies for the individual to master tasks that they may find tedious and uninteresting. For example, thinking of effort as a fluid quality that is malleable and under the control of the individual, rather than fixed and defined by the parameters of the task, may offer promising directions to support students with ADHD who struggle academically. Such an approach is very much in line with current mindset interventions for targeting academic underachievement, such as growth mindset programs (Paunesku et al., 2015).

In this study, individuals at-risk for ADHD rated the task as requiring more effort than the ADHD non-risk group. The group differences in ratings of effort required and discomfort remained significant after controlling for accuracy. For individuals who avoid effortful tasks, there may be a unique affective association that negatively tags their experience of effort, possibly impacting their motivation for future tasks. Given that many theories of mental effort postulate that the experience of being mentally taxed may serve as a regulatory signal to disengage from effortful tasks (e.g., Kurzban, 2016), investigating the experience of mental effort demanded during the completion of a cognitively demanding task in individuals with ADHD-related difficulties would provide a productive line of investigation that could be used to advance our understanding of the regulation of effort exerted in at-risk samples.

## Author Contributions

C-FH contributed to the literature review, summarizing findings and writing up the manuscript. JDE and MT contributed to the development of this article and critical revision of the manuscript for intellectual content. MT and JDE made equal contributions to this paper.

## Conflict of Interest Statement

The authors declare that the research was conducted in the absence of any commercial or financial relationships that could be construed as a potential conflict of interest.
